# Supplementary material for: Association of Asymmetric Dimethylarginine and Diastolic Dysfunction in Patients with Hypertrophic Cardiomyopathy
Source: Biomolecules. 2019 Jul 13;9(7):277. doi: 10.3390/biom9070277 (PMC6681289; doi:10.3390/biom9070277)
Supplement: Supplementary file 1 [file biomolecules-09-00277-s001.pdf]

Table 1. Arginine derivatives in genetically confirmed HCM-patients stratified by diastolic function.

	**HCM No DD (n = 21)**	**HCM Mild DD (n = 10)**	**HCM Moderate/severe DD (n = 23)**	***p*-Value for linear trend**
**ADMA (µmol/L)**	0.62±0.16	0.65±0.10	0.73±0.19	0.035
**SDMA (µmol/L)**	0.47±0.15	0.52±0.27	0.60±0.30	0.090
**hArg (µmol/L)**	1.62±0.44	1.40±0.49	1.58±0.54	0.798

Mean±SD; ANOVA with post test for linear trend. ADMA indicates asymmetric dimethylarginine; Arg, arginine; DD, diastolic dysfunction; hArg, homoarginine; HCM, hypertrophic cardiomyopathy; SD, standard deviation; SDMA, symmetric dimethylarginine

Table 2. Logistic regression analyses for the arginine derivatives and diastolic dysfunction in genetically confirmed HCM-patients (moderate to severe DD vs. normal diastolic function).

	**ADMA**		**SDMA**		**hArg**	
	**OR (95% CI)**	***p*-Value**	**OR (95% CI)**	**P value**	**OR (95% CI)**	***p*-Value**
**Model 1**	2.72 (1.06-6.98)	0.038	1.82 (0.87-3.84)	0.113	0.94 (0.41-2.14)	0.882
**Model 2**	2.23 (0.82-6.06)	0.116	1.74 (0.72-4.16)	0.217	1.18 (0.46-3.03)	0.731
**Model 3**	2.32 (0.75-7.24)	0.146	2.33 (0.75-7.18)	0.142	0.71 (0.22-2.29)	0.565
**Model 4**	2.52 (0.74-8.57)	0.139	2.08 (0.56-7.71)	0.271	0.71 (0.22-2.33)	0.578

Logistic regression analyses with odds ratios (95% CI) per SD increase. Model 1 is unadjusted, model 2 is adjusted for age and sex, model 3 is additionally adjusted for body mass index, diabetes mellitus, coronary artery disease, and arterial hypertension, model 4 is additionally adjusted for estimated glomerular filtration rate. ADMA, asymmetric dimethylarginine; CI, confidence interval; hArg, homoarginine; OR, odds ratio; SD, standard deviation; SDMA, symmetric dimethylarginine

Table 3. Correlation analyses of arginine derivatives with echocardiographic and MRI parameters in HCM patients.

	ADMA						SDMA						Homoarginine					
	**Spearman**		**Pearson**		**Partial correlation**		**Spearman**		**Pearson**		**Partial correlation**		**Spearman**		**Pearson**		**Partial correlation**	
	ρ	***p*-Value**	**ϱ**	***p*-Value**	**ϱ**	***p*-Value**	**ϱ**	***p*-Value**	**ϱ**	***p*-Value**	**ϱ**	***p*-Value**	**ϱ**	***p*-Value**	**ϱ**	***p*-Value**	**ϱ**	***p*-Value**
E wave	0.18	0.008	0.07	0.531	0.06	0.574	0.15	0.032	0.07	0.500	0.10	0.338	-0.03	0.694	-0.01	0.904	0.04	0.743
A wave	0.13	0.072	0.04	0.694	-0.01	0.962	0.12	0.097	0.08	0.440	0.06	0.608	0.01	0.943	-0.01	0.886	-0.01	0.929
E/A	-0.03	0.709	0.02	0.860	0.05	0.659	-0.03	0.652	0.02	0.847	0.03	0.783	-0.01	0.857	-0.04	0.696	-0.04	0.702
Mean E′	-0.19	0.007	-0.04	0.706	0.03	0.779	-0.07	0.325	-0.07	0.492	0.00	0.995	0.17	0.019	0.16	0.117	0.10	0.357
Mean E/E′	0.23	<0.001	0.05	0.652	-0.04	0.727	0.17	0.019	0.08	0.453	0.08	0.468	-0.12	0.097	-0.15	0.140	-0.09	0.420
Mean IVRT	0.09	0.217	0.00	0.967	-0.03	0.813	0.03	0.706	-0.02	0.842	-0.05	0.660	-0.19	0.008	-0.16	0.124	-0.10	0.344
LA diameter	0.12	0.088	-0.02	0.866	-0.01	0.960	-0.08	0.238	-0.18	0.075	-0.10	0.362	-0.02	0.750	0.09	0.394	-0.04	0.705
SW thickness	-0.12	0.073	-0.15	0.145	-0.15	0.154	-0.13	0.061	-0.18	0.092	-0.13	0.240	-0.23	<0.001	-0.10	0.316	-0.18	0.096
LW thickness	0.05	0.517	-0.04	0.669	-0.12	0.286	0.03	0.719	0.04	0.673	-0.03	0.801	0.00	0.966	-0.05	0.628	-0.06	0.579
MCF (%)	-0.03	0,750	0.05	0.661	0.05	0.631	0,05	0,556	-0.08	0.437	-0.14	0.186	-0,02	0,818	-0.03	0.775	-0.01	0.925
LGE size (% LV)	-0.13	0.174	0.03	0.742	0.05	0.614	0.00	0.962	0.17	0.095	0.12	0.266	0.13	0.179	-0.08	0.420	-0.08	0.489

Partial correlation analyses are adjusted for age and sex, body mass index, diabetes mellitus, coronary artery disease, and arterial hypertension, and estimated glomerular filtration rate. ADMA indicates asymmetric dimethylarginine; hArg, homoarginine; HCM, hypertrophic cardiomyopathy; IVRT, isovolumetric relaxation time; LA, left atrium; LGE, late-gadolinium-enhancement; LW, lateral wall; MCF, myocardial contraction fraction; SDMA, symmetric dimethylarginine; SW, septal wall

Table 4. Linear regression analyses of arginine derivatives (independent variables) with echocardiographic parameters of diastolic function (dependent variables).

	ADMA		SDMA		hArg	
	**Beta coefficient (95% CI)**	***p*-Value**	**Beta coefficient (95% CI)**	***P* value**	**Beta coefficient (95% CI)**	***p*-Value**
E wave						
**Model 1**	4.90 (0.79-9.01)	0.020	3.61 (-0.36-7.57)	0.075	-0.24 (-4.24-3.75)	0.904
**Model 2**	4.58 (0.35-8.80)	0.034	2.91 (-1.27-7.09)	0.172	1.57 (-2.51-5.65)	0.448
**Model 3**	4.72 (0.43-9.01)	0.031	3.14 (-1.10-7.39)	0.146	1.39 (-2.83-5.60)	0.518
**Model 4**	4.67 (0.37-8.98)	0.034	4.10 (-0.41-8.61)	0.074	1.40 (-2.84-5.65)	0.515
A wave						
**Model 1**	0.83 (-6.97-8.64)	0.833	4.00 (-4.20-12.19)	0.338	0.34 (-7.26-7.95)	0.929
**Model 2**	-2.78 (-10.74-5.18)	0.492	-0.33 (-8.88-8.23)	0.940	1.32 (-6.37-9.00)	0.735
**Model 3**	-2.15 (-10.09-5.78)	0.593	-0.51 (-9.09-8.07)	0.907	-0.19 (-7.98-7.60)	0.962
**Model 4**	-2.18 (-10.15-5.79)	0.590	0.79 (-8.30-9.87)	0.865	-0.20 (-8.04-7.64)	0.959
E/A						
**Model 1**	0.05 (-0.05-0.15)	0.284	0.00 (-0.11-0.10)	0.948	-0.05 (-0.14-0.05)	0.330
**Model 2**	0.10 (0.00-0.20)	0.053	0.05 (-0.06-0.16)	0.402	-0.04 (-0.14-0.05)	0.367
**Model 3**	0.09 (-0.01-0.20)	0.067	0.05 (-0.06-0.16)	0.404	-0.02 (-0.12-0.08)	0.657
**Model 4**	0.10 (0.00-0.20)	0.061	0.02 (-0.09-0.14)	0.689	-0.02 (-0.12-0.08)	0.684
Mean E′						
**Model 1**	-0.36 (-0.68--0.05)	0.024	-0.19 (-0.50-0.12)	0.237	0.21 (-0.10-0.53)	0.188
**Model 2**	-0.14 (-0.44-0.17)	0.378	0.13 (-0.17-0.43)	0.396	0.00 (-0.30-0.30)	0.993
**Model 3**	-0.16 (-0.46-0.14)	0.303	0.10 (-0.20-0.40)	0.519	0.11 (-0.20-0.41)	0.491
**Model 4**	-0.17 (-0.47-0.13)	0.272	0.12 (-0.21-0.44)	0.475	0.09 (-0.21-0.39)	0.557
Mean E/E′						
**Model 1**	2.24 (1.17-3.31)	<0.001	0.85 (-0.23-1.92)	0.122	-0.24 (-1.34-0.87)	0.673
**Model 2**	1.80 (0.77-2.83)	<0.001	0.10 (-0.96-1.16)	0.854	0.67 (-0.38-1.73)	0.208
**Model 3**	1.76 (0.73-2.79)	<0.001	0.10 (-0.96-1.17)	0.850	0.49 (-0.57-1.55)	0.362
**Model 4**	1.77 (0.74-2.80)	<0.001	0.36 (-0.77-1.49)	0.532	0.48 (-0.59-1.54)	0.379
Mean IVRT						
**Model 1**	2.32 (-2.66-7.31)	0.359	-0.60 (-5.45-4.25)	0.808	-4.03 (-8.94-0.89)	0.108
**Model 2**	0.05 (-5.07-5.16)	0.985	-3.79 (-8.85-1.26)	0.140	-3.16 (-8.18-1.86)	0.215
**Model 3**	0.20 (-5.01-5.41)	0.941	-3.63 (-8.80-1.53)	0.167	-3.82 (-8.96-1.32)	0.144
**Model 4**	0.18 (-5.07-5.42)	0.947	-4.34 (-9.84-1.16)	0.121	-3.87 (-9.07-1.33)	0.144
LA diameter						
**Model 1**	0.78 (-0.67-2.23)	0.289	0.40 (-1.00-1.80)	0.573	-0.32 (-1.75-1.11)	0.660
**Model 2**	0.21 (-1.29-1.70)	0.786	-0.29 (-1.77-1.18)	0.697	-0.32 (-1.78-1.15)	0.672
**Model 3**	0.76 (-0.71-2.23)	0.307	0.30 (-1.15-1.75)	0.685	-0.76 (-2.21-0.69)	0.304
**Model 4**	0.87 (-0.60-2.33)	0.244	0.05 (-1.48-1.58)	0.948	-0.58 (-2.03-0.88)	0.435

Linear regression analyses with beta coefficients (95% CI) per SD Arg derivatives increase. Model 1 is unadjusted, model 2 is adjusted for age and sex, model 3 is additionally adjusted for body mass index, diabetes mellitus, coronary artery disease, and arterial hypertension, model 4 is additionally adjusted for estimated glomerular filtration rate. ADMA, asymmetric dimethylarginine; CI, confidence interval; hArg, homoarginine; IVRT, isovolumetric relaxation time; LA, left atrium; SD, standard deviation; SDMA, symmetric dimethylarginine.

Table 5. Logistic regression analyses of arginine derivatives and atrial fibrillation in genetically confirmed HCM-patients.

	ADMA		SDMA		hArg	
	**OR (95% CI)**	***p*-Value**	**OR (95% CI)**	***p*-Value**	**OR (95% CI)**	***p*-Value**
**Model 1**	1.85 (0.85-4.05)	0.123	2.00 (1.05-3.79)	0.034	0.53 (0.23-1.23)	0.137
**Model 2**	1.64 (0.70-3.83)	0.256	2.00 (0.97-4.13)	0.060	0.60 (0.25-1.45)	0.260
**Model 3**	1.63 (0.64-4.18)	0.306	2.06 (0.86-4.92)	0.105	0.44 (0.16-1.25)	0.125
**Model 4**	1.67 (0.65-4.29)	0.289	1.78 (0.70-4.50)	0.223	0.51 (0.18-1.48)	0.215

Logistic regression analyses with odds ratios (95% CI) per SD increase. Model 1 is unadjusted, model 2 is adjusted for age and sex, model 3 is additionally adjusted for body mass index, diabetes mellitus, coronary artery disease, and arterial hypertension, model 4 is additionally adjusted for estimated glomerular filtration rate. ADMA, asymmetric dimethylarginine; CI, confidence interval; hArg, homoarginine; OR, odds ratio; SD, standard deviation; SDMA, symmetric dimethylarginine.
